# Liver transplantation and comprehensive rehabilitation in the reversal of hepatic myelopathy: a case report

**DOI:** 10.3389/fmed.2024.1467611

**Published:** 2024-11-12

**Authors:** Mingyue Liu, Rongping Wang, Wenwen Zhou, Chunyan Tian, Qian Wang, Qin Zhang, Shasha Jin, Liang Wu

**Affiliations:** ^1^Department of Sports Rehabilitation, Beijing Xiaotangshan Hospital, Beijing, China; ^2^Medical Imaging Department, Beijing Xiaotangshan Hospital, Beijing, China; ^3^Department of Physical Therapy, Beijing Xiaotangshan Hospital, Beijing, China

**Keywords:** hepatic myelopathy, spastic paraplegia, portosystemic shunt, liver transplantation, rehabilitation

## Abstract

Hepatic myelopathy (HM) is a rare complication of end-stage chronic liver disease, primarily presenting as symmetrical lower limb weakness that progresses to spastic paralysis without sensory or sphincter dysfunction. We report a case of decompensated cirrhosis associated with hepatitis B virus and HM. The patient showed significant recovery after liver transplantation (LT) and comprehensive rehabilitation training. We summarize the patient’s clinical characteristics post-diagnosis and the assessment outcomes following integrated treatment.

## 1 Introduction

Hepatic myelopathy (HM) was first reported in 1949 (1). It is a rare complication of the central nervous system associated with the terminal stage of chronic liver disease, primarily caused by alcoholic cirrhosis and hepatitis B cirrhosis. The occurrence of HM following acute fulminant hepatic failure is infrequent (2). In most documented cases, HM presents as spastic paraplegia of the lower limbs, with rare instances of tetraplegia. Physical examination typically reveals increased muscle tone and hyperreflexia in the lower limbs, without sensory or sphincter dysfunction (3). Research indicates that males, and patients who have undergone splenectomy, splenorenal shunting, or transjugular intrahepatic portosystemic shunt (TIPS), especially those with recurrent episodes of hepatic encephalopathy, are at higher risk for developing HM (4). The onset of HM is often insidious, and the diagnosis is primarily exclusionary, necessitating the elimination of various structural and primary neurological disorders in the context of a highly suspicious clinical presentation. Conservative treatment with ammonia-lowering agents and other medications has shown limited effectiveness, emphasizing the importance of liver transplantation for early-stage patients (5, 6). Here, we present a case of an adult male who developed lower limb weakness 5 months post-TIPS placement and showed a favorable recovery following liver transplantation (LT) and comprehensive rehabilitation training.

## 2 Case presentation

**Figure 1:**
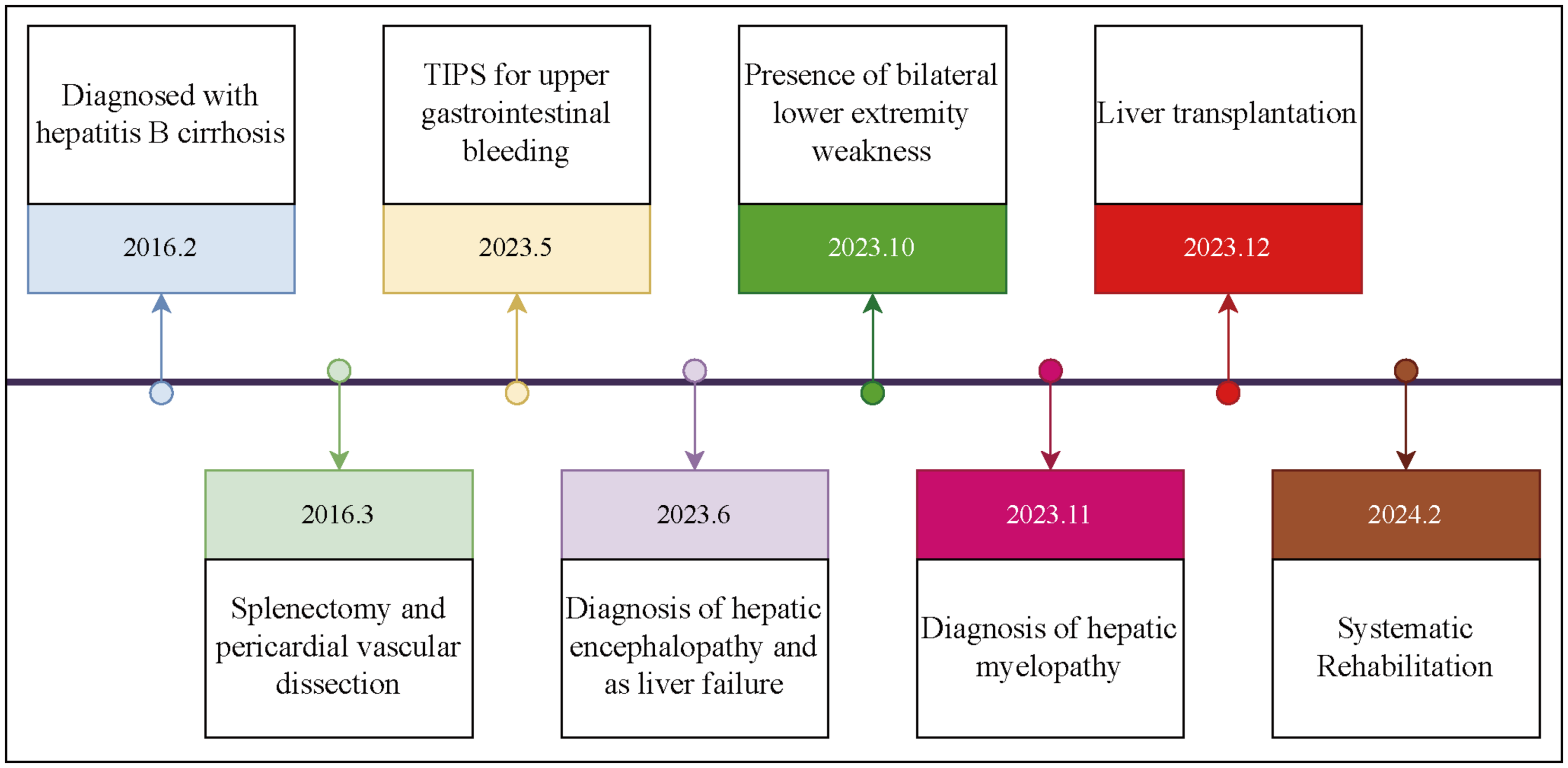
Course of the patient from the diagnosis of liver cirrhosis to the initiation of treatment.

The patient was a 35-year-old Chinese (Asian) male who initially presented on February 1, 2016, with a 1-week history of abdominal distension. He was diagnosed with hepatitis B cirrhosis after testing positive for HBsAg. On March 1, 2016, the patient presented with complaints of fatigue and gum bleeding. Upon admission, laboratory investigations revealed a white blood cell count of 2.14 × 10^9^/L (reference range: 4.0–10.0 × 10^9^/L), a hemoglobin concentration of 124 g/L (reference range: 120–160 g/L), and a platelet count of 37 × 10^9^/L (reference range: 100–300 × 10^9^/L). Gastrointestinal endoscopy identified multiple esophageal varices situated 25 cm from the incisors, in addition to a tumor-like varix in the gastric fundus. Ultrasonographic evaluation demonstrated splenomegaly, with a longitudinal diameter of 177 mm, splenic hilum thickness of 63 mm, and a splenic vein diameter of 16 mm. On the sixth day of hospitalization, the patient underwent splenectomy and esophagogastric devascularization under general anesthesia. Following discharge, he was prescribed antiviral therapy and hepatoprotective medications. Despite these treatments, the patient experienced recurrent complications, including persistent episodes of diarrhea, gum bleeding, epistaxis, pulmonary infections, and pleural effusions. On May 9, 2023, the patient was admitted to the emergency department due to hematemesis-induced coma. Upon admission, vital signs indicated severe hypotension with a blood pressure of 76/39 mmHg, and laboratory tests revealed critically low hemoglobin levels of 47 g/L. Abdominal computed tomography (CT) imaging suggested cirrhosis, characterized by a linear hyperdense signal between the liver and stomach. The clinical diagnosis was esophagogastric variceal hemorrhage secondary to cirrhosis. The patient underwent urgent interventions, including transjugular gastric variceal embolization and TIPS placement. During hospitalization in June 2023, he exhibited recurrent cognitive decline, leading to a diagnosis of hepatic encephalopathy. The patient also showed signs of poor mental status, with multiple episodes of prothrombin activity below 40%, indicating liver failure. On October 5, 2023, he developed stiffness while walking, which progressively worsened without significant sensory impairment in the lower limbs. By November 2023, these symptoms worsened, accompanied by pitting edema in the lower limbs. He was subsequently admitted to the hepatobiliary surgery department of a hospital in Beijing, where he was diagnosed with HM. Upon admission, his blood ammonia level was 132.5 μmol/L (normal range: 10–47 μmol/L). Neurological examination revealed spastic paraplegia in the lower limbs with muscle strength at grade 2, increased muscle tone, and an inability to stand independently; upper limb function was normal. On December 1, 2023, after completing relevant examinations, the patient underwent LT from a brain-dead donor. One month postoperatively, the patient still exhibited difficulty lifting his legs from the bed, but the rigidity had decreased. The progression of the patient’s condition since the diagnosis of cirrhosis is illustrated in Figure 1.

**Figure 2:**
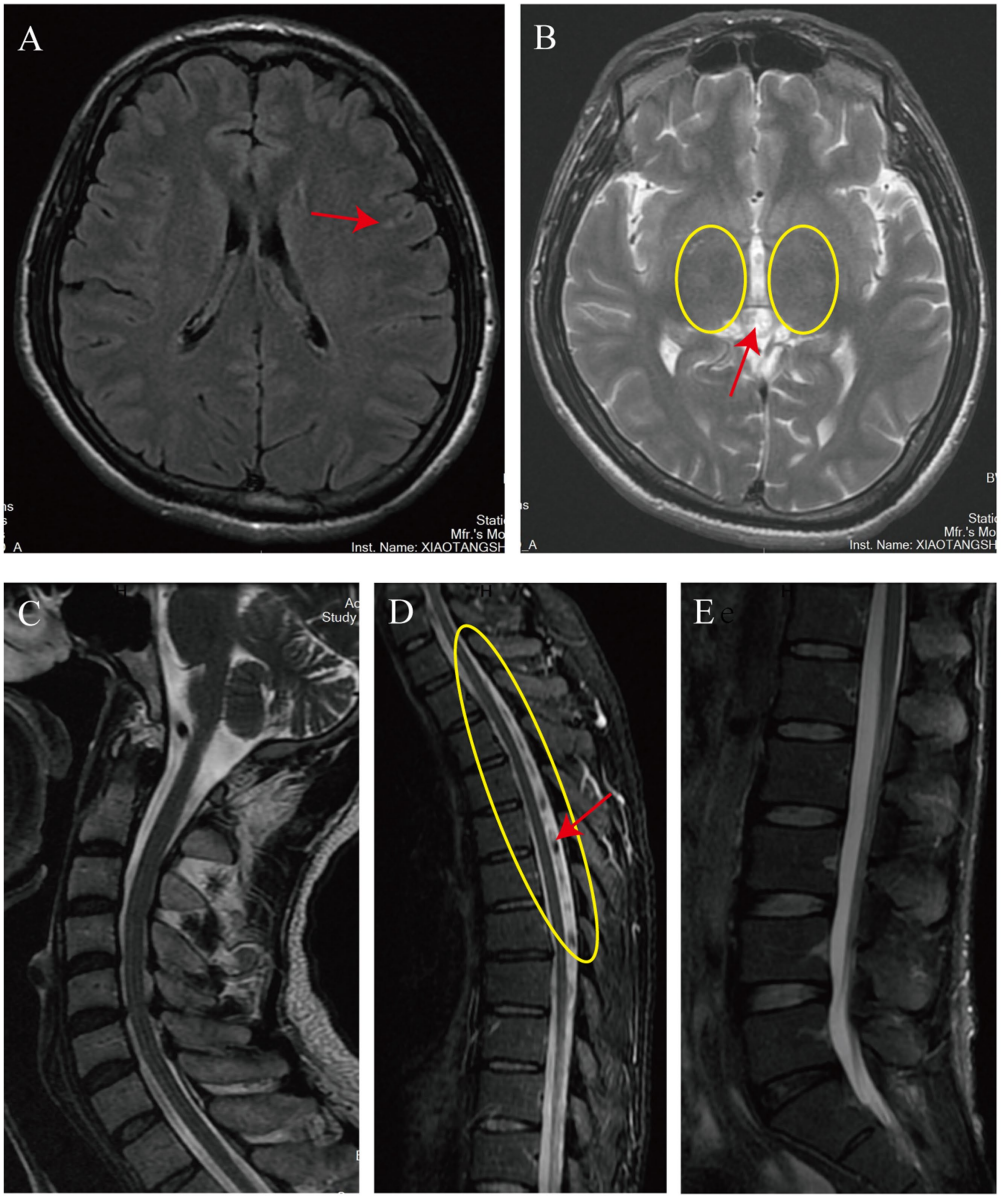
The imaging findings upon the patient’s admission. The cranial MRI reveals patchy T2-FLAIR hyperintense signals in the left frontal lobe (indicated by the red arrow in **A**). Additionally, it shows faintly hyperintense, slightly increased signals in the bilateral basal ganglia on T2-weighted images (encircled in yellow in **B**) and an isointense pineal gland with a diameter of approximately 11 mm (also indicated by the red arrow in **A**). The MRI of the entire cervical and lumbar spinal cord shows no significant abnormalities **(C,E)**. However, the thoracic spine MRI demonstrates a tortuous and thickened capillary plexus within the dural sac on T2-weighted images from T2 to T8 (encircled in yellow in **D**), with some vessels exhibiting a poor flow void effect (indicated by the red arrow in **D**).

**Figure 3:**
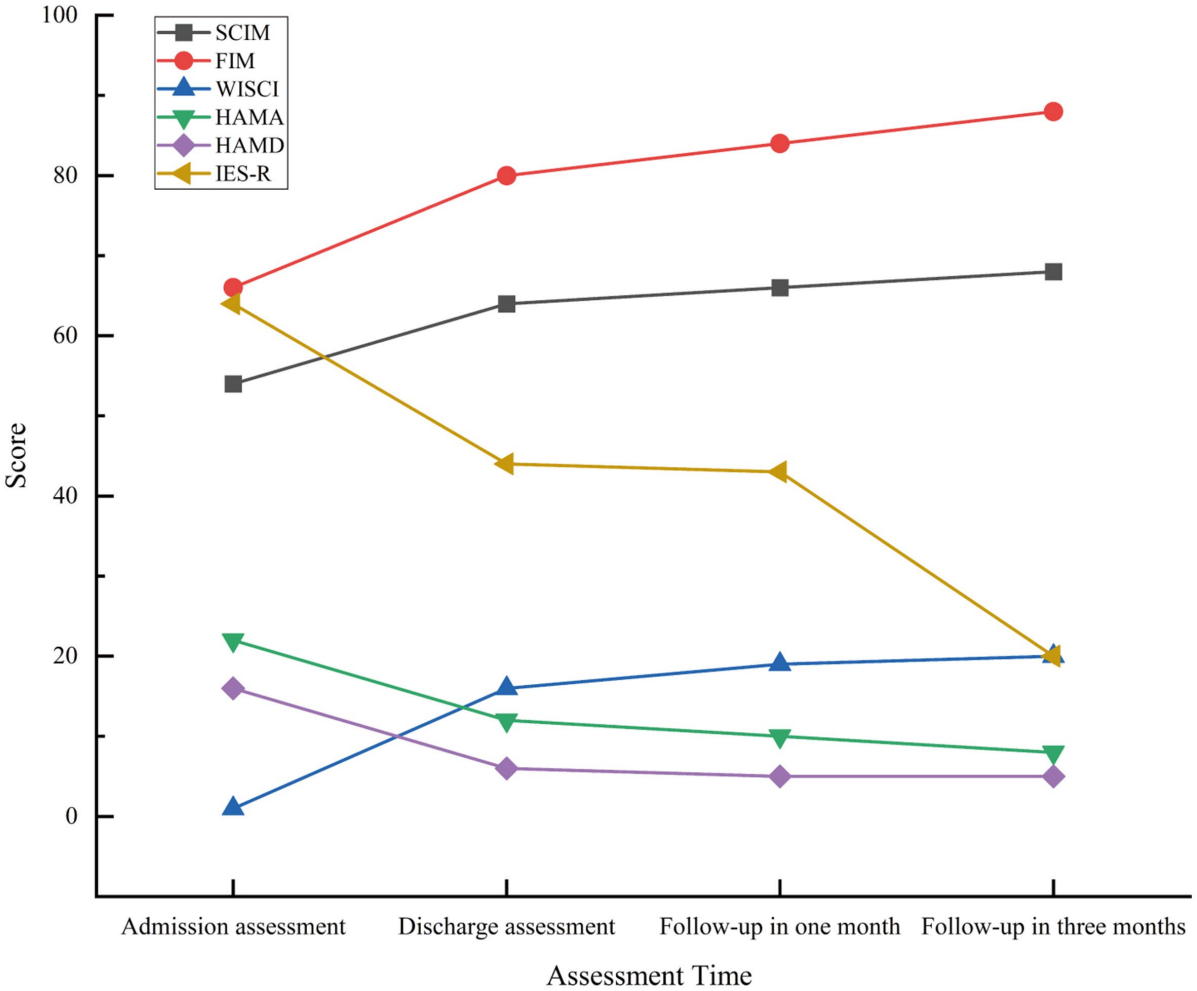
Patient scale assessment results, including admission and follow-up evaluations.

On February 3, 2024, the patient presented to our department of movement rehabilitation with difficulty walking, decreased muscle strength in the lower limbs, and abnormally increased muscle tone. Upon admission, a neurological examination revealed spastic paraplegia, with the ability to walk with 90% weight-bearing status using a scissoring gait. The muscle strength in the lower limbs was grade 3, accompanied by hyperactive deep tendon reflexes and positive bilateral Babinski signs. There were no superficial or deep sensory disturbances, nor symptoms of intestinal or bladder involvement. Cranial nerve examination was normal. The patient’s liver function and total bilirubin levels were within the normal range, with detailed hematological and biochemical characteristics shown in Table 1. Brain MRI showed lesions in the frontal lobe and bilateral basal ganglia, suggesting degeneration (Figures 2A,B). MRI of the cervical and lumbar spine showed no significant abnormalities, but MRI of the thoracic spine (T2–T8) revealed thickening and tortuosity of the capillary plexus within the dural sac, with poor flow void effect in some vessels (Figures 2C–E). Motor evoked potentials showed no significant abnormalities, while somatosensory evoked potentials indicated delayed deep sensory conduction in the lower limbs, with no other abnormalities. In terms of treatment, the patient received comprehensive rehabilitation training while maintaining anti-rejection and antiviral therapies. This included physical therapy (PT) for paraplegic patients, psychological counseling, social and vocational rehabilitation, respiratory training, neuromuscular electrical stimulation, peripheral repetitive magnetic stimulation, aquatic exercises, and acupuncture treatment. The patient was discharged after 40 days and continued with weekly remote rehabilitation guidance training. Assessments using relevant scales were conducted before and after rehabilitation treatment, and at the first and third months of follow-up. These included the Spinal Cord Injury Motor Score (SCIM), Functional Independence Measure (FIM), Walking Index for Spinal Cord Injury (WISCI), Hamilton Anxiety Scale (HAMA), Hamilton Depression Scale (HAMD), and the Impact of Event Scale-Revised (IES-R). The assessment results are shown in Figure 3. 6 month post-transplantation, the patient’s liver function and total bilirubin levels remained normal. The muscle strength in the lower limbs improved to grade 4, allowing for independent walking of approximately 50 m, with an improved scissoring gait compared to before. No other neurological symptoms were reported during the follow-up period.

**Table 1:** Hematological, biochemical, and nutritional characteristics of the patient at key time points.

Test item (Unit)	Splenectomy	TIPS	LT	Rehabilitation	Reference range
H/W (cm/kg)	184/NA	184/70	184/74	184/69	NA
BMI (kg/m^2^)	NA	20.7	21.9	20.4	18.5–23.9
ALT (U/L)	31.1	30.1	25.4	13.0	9–50
AST (U/L)	24.9	39.7	52.1	18.3	15–40
ALP (g/L)	NA	64.7	94.1	40.5	40–55
GGT (U/L)	NA	24.5	26.0	15.5	0–125
CHE (U/L)	NA	1,689	1944	10,050	5,320–12,920
TBIL (μmol/L)	32.1	45.8	73.9	7.3	17–23
DBIL (μmol/L)	19.2	21.1	45.7	3.8	0–8
TBA (μmol/L)	NA	14.6	230.0	7.2	0–10
TP (g/L)	61.4	49.0	55.9	60.8	65–85
ALB (g/L)	36.3	20.8	29.5	45.2	35–52
GLB (g/L)	25.1	28.2	26.5	31.6	20–35
PREA (mg/dL)	NA	NA	0.04	0.31	0.2–0.34
TRIG (mmol/L)	0.7	1.0	0.7	0.9	0–1.7
CHOL (mmol/L)	3.1	3.3	3.1	4.1	0–5.2
HDL-C	1.60	1.40	1.12	1.78	0.77–2.25
LDL-C	1.07	1.30	1.54	2.35	0–3.36
Apo-A1 (mg/dL)	NA	0.77	1.10	1.43	1.2–1.6
Apo-B (mg/dL)	NA	0.55	0.51	0.89	0.8–1.1
sd LDL-C (mmol/L)	NA	0.11	0.14	0.66	0.246–1.393
PT (s)	11.5	17.7	12.1	12.6	10–13
APTT (s)	23.1	65.4	25.5	26.1	23.3–32.5
FIB (g/L)	2.9	1.05	3.62	1.91	1.8–3.5
Mn (μmol/L)	NA	NA	NA	Normal	0–255
Vitamin B1 (ng/mL)	NA	NA	Normal	Normal	80–200
Vitamin B6 (nmol/L)	NA	NA	Normal	Normal	20–200
Vitamin B12 (pmol/L)	NA	NA	Normal	Normal	133–675
25-HD (nmol/L)	NA	Normal	Normal	16	25–150

H/W, Height/Weight; BMI, Body mass index; ALT, Alanine aminotransferase; AST, Aspartate aminotransferase; ALP, Alkaline phosphatase; GGT, Gamma-glutamyl transferase; CHE, Cholinesterase; TBIL, Total bilirubin; DBIL, Direct bilirubin; TBA, Total bile acid; TP, Total protein; ALB, Albumin; GLB, Globulin; PREA, Prealbumin; TRIG, Triglycerides; CHOL, Cholesterol; HDL-C, High-density lipoprotein cholesterol; LDL-C, Low-density lipoprotein cholesterol; Apo-A1, Apolipoprotein AI; Apo-B, Apolipoprotein B; sd LDL-C, Small dense low-density lipoprotein cholesterol; PT, Prothrombin time; APTT, Activated partial thromboplastin time; FIB, Fibrinogen; Mn, Manganese; Vitamin B1, Thiamine; Vitamin B6, Pyridoxine; Vitamin B12, Cobalamin; 25-HD, 25-Hydroxyvitamin D; NA, Not available; s, second.

## 3 Discussion

Hepatic myelopathy is a relatively rare neurologic complication associated with advanced liver disease, often underdiagnosed due to its low incidence in clinical practice. It is commonly believed to be related to the spontaneous formation of portal-systemic shunts or surgeries performed on patients with liver disease. The characteristic features of HM include bilateral, chronic, progressive, symmetric spastic paraplegia, with rare involvement of the sphincters and sensory systems, and severe myopathy accompanied by extensive portal collateral circulation (7, 8). In this case, the patient developed HM 5 months after the TIPS procedure, exhibiting clinical symptoms similar to those previously reported.

The exact pathogenesis of HM is not fully understood due to the lack of a mature animal model to simulate the disease process. Current research suggests that the onset of HM may involve multiple pathophysiological mechanisms, including toxic damage, nutritional deficiency, hemodynamic changes, and immune damage (9). The toxic damage mechanism posits that ammonia and other nitrogenous toxins accumulate when liver function is impaired, crossing the blood–brain barrier and causing neuronal damage, potentially leading to symmetrical demyelination of the corticospinal tracts, which is one of the early spinal cord injury characteristics of HM (10). In this case, the patient had multiple episodes of elevated blood ammonia levels before LT, likely contributing to the progression of HM. The lesions may also involve the giant Betz cells in the internal layer of the precentral gyrus of the cerebral cortex, which are the nutritional centers of the corticospinal tracts. This can lead to gradual degeneration and loss of nerve fibers from the distal to the proximal end, with severe cases extending to the brainstem and internal capsules, ultimately causing irreversible spinal cord nerve tissue damage. The nutritional deficiency mechanism emphasizes the impact of liver insufficiency on the absorption and synthesis of neuroprotective substances. Deficiencies in vitamins, phospholipids, and other substances may lead to a lack of proteins and enzymes in spinal cord nerve fiber cells, thereby affecting the synthesis and renewal of neurotransmitters (11). In this case, the blood ammonia, manganese ions, and vitamin levels checked after admission were within the normal range, likely due to the recovery of liver function after liver transplantation. However, due to the lack of these indicators before liver transplantation, it is not possible to exclude whether these factors impacted the progression of the patient’s HM. The hemodynamic change mechanism focuses on the potential impact of portal hypertension and its spontaneous shunting phenomenon on the onset of HM. Long-term portal hypertension may lead to chronic ischemia and hypoxia of the thoracolumbar spinal cord, especially in areas with obvious segmental characteristics of blood supply, such as the abdominal lateral wall of the fourth thoracic and first lumbar segments, where spinal cord damage is particularly obvious (12). In this case, the thoracic spine MRI at the T2–T8 segment showed tortuous and thickened capillary plexus within the dural sac, with poor flow void effect in some vessels, indicating poor blood flow in this segment of the spinal cord. This is likely to lead to chronic ischemia and hypoxia of this segment of the spinal cord, resulting in corresponding clinical symptoms. The immune damage mechanism suggests that chronic liver disease, mainly caused by viral infections, may cause spinal cord nerve damage through the deposition of immune complexes in the nervous system. In summary, the pathogenesis of HM is the result of multiple factors and mechanisms acting together. Future research needs to further explore the interaction between these mechanisms and how they jointly affect the pathological process of HM. Additionally, the development of effective animal models is crucial for a deeper understanding of the pathogenesis of HM and for finding potential treatment methods.

The diagnosis of HM requires the exclusion of other causes of spastic paraplegia, such as subacute combined degeneration of the spinal cord caused by vitamin B12 deficiency, hepatolenticular degeneration, primary lateral sclerosis, multiple sclerosis, and hereditary spastic paraplegia. Laboratory biochemical test results usually relate to liver dysfunction or cirrhosis, such as increased blood ammonia levels, abnormal liver function indicators, and abnormal coagulation function (13). Cranial MRI in patients with HM may show high signals in the basal ganglia on T1-weighted imaging (14). In this case, abnormal signals in the frontal lobe and bilateral basal ganglia may be related to the aforementioned pathogenesis, but it cannot be excluded that they are related to the patient’s history of syncope and ischemia of the cortex and deep nuclei caused by hepatic encephalopathy. In most cases, head imaging examinations of patients with HM do not show obvious abnormalities. In terms of electrophysiology, Nardone et al. showed that the central motor conduction time (CMCT) of patients with HM was prolonged, which is a key indicator of the conduction time from motor cortical neurons to spinal motor neurons. Its prolongation reflects damage to the corticospinal tract, the main nerve fiber bundle controlling voluntary movement of skeletal muscles. This damage may be the main reason for the motor disorders of patients with HM. The study also pointed out that before the patient develops sensory disorders, the damage to the sensory pathway can be assessed by electrophysiological assessment of central sensory conduction (15). These findings emphasize the importance of electrophysiological testing in the early diagnosis of HM, especially in the preclinical stage. A significant increase in CMCT may indicate a reduction in the recovery of HM nerve function. In this case, there was a lack of corresponding electrophysiological testing in the early stage, and the relevant electrophysiological testing after admission showed no obvious abnormalities in CMCT, which may provide the possibility for the patient’s further recovery. Although the patient’s physical examination showed no obvious sensory abnormalities, the somatosensory evoked potential test showed delayed conduction in the deep sensory conduction pathway of the lower limbs, which may also be an important factor affecting the patient’s balance function.

At present, there is no proven effective treatment for HM. Existing literature indicates that LT can significantly alleviate the clinical symptoms of patients with HM and may be effective (5, 6, 16), but the effect of LT on patients with advanced HM is not obvious (17). Timely control of blood ammonia may prevent the further development of HM and improve the patient’s prognosis (7). There are reports that embolization of some parts of the splenic artery can alleviate severe spastic HM, thereby greatly reversing the severe spastic paraplegia caused by HM (18). Sun et al. were the first to apply fecal microbiota transplantation to treat HM (19). In this case, the patient underwent systematic rehabilitation training after LT, including a long-term plan for subsequent remote rehabilitation, while emphasizing the repair of psychological trauma during the rehabilitation process. This included the use of cognitive therapy, spiritual reshaping therapy, and music therapy. In comprehensive rehabilitation training, the patient achieved good benefits in both psychological and life ability aspects, providing some reference for future cases.

## 4 Conclusion

In summary, HM typically presents clinically as a chronic, progressive spastic paraplegia of the lower limbs. However, the underlying pathophysiology remains elusive. Specific biochemical markers, electrophysiological assessments of central sensory-motor conduction, and thoracic spinal MRI hold significant potential for aiding in the diagnosis and prognostication of HM. LT combined with comprehensive rehabilitation protocols has been proposed as a potentially effective therapeutic approach. Nevertheless, current literature on this condition is sparse, and standardized rehabilitation protocols for HM are lacking, underscoring the need for further prospective studies to validate these findings. Moreover, innovative treatment modalities, such as advanced surgical techniques and fecal microbiota transplantation, warrant further investigation.

## Data Availability

The original contributions presented in the study are included in the article/supplementary material, further inquiries can be directed to the corresponding authors.
